# High-Performance Edge-Contact Monolayer Molybdenum Disulfide Transistors

**DOI:** 10.34133/research.0593

**Published:** 2025-01-17

**Authors:** Jiankun Xiao, Xiong Xiong, Xinhang Shi, Shiyuan Liu, Shenwu Zhu, Yue Zhang, Ru Huang, Yanqing Wu

**Affiliations:** ^1^ School of Integrated Circuits and Beijing Advanced Innovation Center for Integrated Circuits, Peking University, Beijing 100871, China.; ^2^ Wuhan National High Magnetic Field Center and School of Integrated Circuits, Huazhong University of Science and Technology, Wuhan 430074, China.; ^3^ Academy for Advanced Interdisciplinary Science and Technology, Beijing Advanced Innovation Center for Materials Genome Engineering, University of Science and Technology Beijing, Beijing 100083, China.

## Abstract

Edge contact is essential for achieving the ultimate device pitch scaling of stacked nanosheet transistors with monolayer 2-dimensional (2D) channels. However, due to large edge-contact resistance between 2D channels and contact metal, there is currently a lack of high-performance edge-contact device technology for 2D material channels. Here, we report high-performance edge-contact monolayer molybdenum disulfide (MoS_2_) field-effect transistors (FETs) utilizing well-controlled plasma etching techniques. Plasma etching with pure argon improves the edge dangling bonds and thus improves the edge-contact quality. Edge-contact monolayer MoS_2_ FET shows good ohmic contact even at cryogenic temperatures (20 K), achieving a record-low contact resistance (*R*_c_) of 1.25 kΩ·μm among all edge-contact MoS_2_ devices. The record-high on-state current of 436 μA/μm and transconductance of 123 μS/μm at *V*_ds_ = 1 V are achieved on an edge-contact monolayer MoS_2_ FET with *L*_ch_ = 120 nm. This work highlights the great potential of edge contacts for high-performance monolayer transition metal dichalcogenide (TMD) material electronics.

## Introduction

**Fig. 1.**
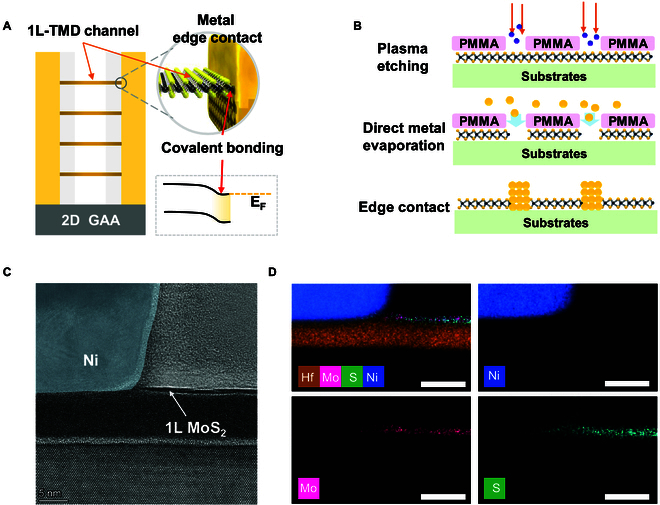
Schematic diagram and characterization of edge-contact structure. (A) Schematic diagram of 2D gate-all-around (GAA) nanosheet device constructed by edge-contact structure. (B) Schematic diagram of key process flow for monolayer MoS_2_ edge-contact FET fabrication. (C) Scanning transmission electron microscopy image of edge contact between metal Ni and the edge of monolayer MoS_2_. Scale bar, 5 nm. (D) STEM-EDX elemental map of edge contact between metal Ni and the edge of monolayer MoS_2_. The elemental map of metal Ni is connected to those of Mo and S between metal Ni and the edge of monolayer MoS_2_. Scale bars, 10 nm.

The continuous scaling of traditional semiconductor electronics has faced increasing challenges, especially in terms of surging power consumption and more severe short channel effects. In recent years, the emergence of 2-dimensional (2D) transition metal dichalcogenide (TMD) materials has attracted widespread attention, particularly for the next generation of electronic device architectures for multi-stacked nanosheet devices, owing to their high mobility and excellent electrostatic control at atomically thin thicknesses of below 1 nm [1–6]. Both experimental and theoretical studies have demonstrated and verified the great potential of TMD materials in constructing ultra-short-channel field-effect transistors (FETs) [7–10]. Forming high-quality ohmic contacts is a prerequisite for the future application of monolayer TMD channel FETs [11–15]. The most common and convenient approach is to directly deposit metals onto monolayer TMD materials to form top contacts [16–25]. To date, depositing semi-metals such as antimony and bismuth onto monolayer molybdenum disulfide (MoS_2_) with a top contact structure has resulted in a low contact resistance approaching the quantum limit [26,27]. In addition to top contacts, edge contacts have also attracted widespread interest due to their natural advantages in reducing contact length and enabling the construction of vertically stacked nanosheet FETs for ultimate device pitch scaling (Fig. 1A) [28–35]. Previous studies have shown that edge contacts on MoS_2_ FETs can eliminate Fermi pinning compared to the common top contacts, attributed to the formation of covalent bonds between 2D MoS_2_ and metals presenting a short-range dipole interface [36,37]. However, limited by the high contact resistance for edge contacts, key metrics of high-performance devices, such as on-state current and transconductance, remain lower than expected. Therefore, the realization of high-performance edge-contact monolayer MoS_2_ FETs is imperative.

In this work, high-quality edge-contacted FETs are realized using monolayer MoS_2_ grown by chemical vapor deposition (CVD), which can be clearly verified from the transmission electron microscopy (TEM) image. By using well-controlled plasma etching technology to form clean edge-contact regions, the etched areas are less susceptible to various molecules, free radicals, adsorbates, and air exposure generated during the plasma process. Specifically, nickel/gold (Ni/Au) contacts were evaporated under high vacuum at a slow deposition rate to serve as ohmic contacts. The edge-contact monolayer MoS_2_ FETs exhibited a linear current–voltage relationship at a cryogenic temperature of 20 K. The extracted Schottky barrier of the edge-contact devices was as low as 32 meV, and the contact resistance was 1.25 kΩ·μm. Furthermore, the edge-contact monolayer MoS_2_ FETs with *L*_ch_ = 120 nm on HfO_2_ dielectric demonstrated high on-state current of 436 μA/μm and transconductance of 123 μS/μm at *V*_ds_ = 1 V, far exceeding previously reported edge-contact 2D devices.

## Results

The manufacturing process of the edge-contact FET is depicted in Fig. 1B. Monolayer MoS_2_ film was grown by using CVD method on a glass substrate [38]. The as-grown monolayer MoS_2_ films were then transferred onto an HfO_2_/Si substrate using polymethyl methacrylate (PMMA) as an adhesion/supporting layer by a wet transfer method. Moreover, it is crucial to note that the as-grown high-quality monolayer MoS_2_ is a prerequisite for obtaining high-performance devices [39–41]. The isolation region was etched using inductively coupled plasma (ICP) to obtain a long ribbon of monolayer MoS_2_. An annealing process was conducted to eliminate organic residues to achieve a high-quality monolayer MoS_2_ channel. The contact regions of FETs were patterned using electron beam lithography, while the channel MoS_2_ regions were covered with a PMMA photoresist. Before depositing the contact metal, monolayer MoS_2_ of the contact regions was etched by pure argon (Ar) plasma [the control group was treated with oxygen/argon (O_2_/Ar) plasma, which will be discussed later]. More importantly, the entire etching process was carried out in a clean room with relatively low humidity, and contact metal was deposited immediately after the etching process. This process is to avoid the reaction of numerous dangling bonds (Mo-bonds and S-bonds) of MoS_2_ in the contact regions with various molecules, free radicals, oxygen, and steam in the air to form molybdenum oxide, which reduces the quality of the edge contact [42], which will be further discussed in the following sections. The source/drain contact metal of Ni/Au is evaporated under a high vacuum of 2 × 10^−7^ torr at a slow rate of 0.01 nm/s, because the low damage metal deposition under high vacuum is also a prerequisite for obtaining high-quality edge contact [43,44]. See Fig. S1 for device fabrication process flow.

To further verify the monolayer MoS_2_ edge-contact structure more intuitively, the cross-sectional TEM characterization of the local contact regions of MoS_2_/Ni was performed, as shown in Fig. 1C. It is evident that the successful construction of monolayer MoS_2_ edge contact has been achieved. The edges of the monolayer MoS_2_ on a 9-nm-thick HfO_2_ substrate are connected to the deposited metal Ni, visually indicating the formation of edge contact. The corresponding energy-dispersive x-ray spectroscopy (EDXS) mapping shows the element distribution, and the elemental map of metal Ni is connected to those of Mo and S between metal Ni and the edge of the etched monolayer MoS_2_, proving the formation of edge contact, as shown in Fig. 1D.

**Fig. 2.**
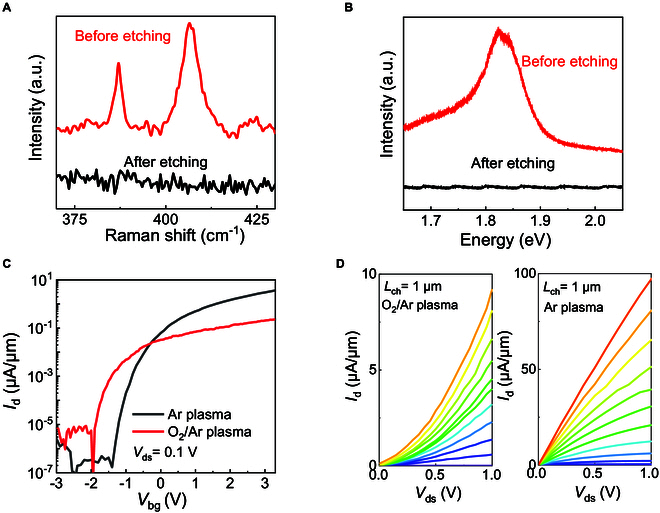
The electrical characteristics of monolayer MoS_2_ edge-contact FETs under different etching conditions. (A) Raman spectra and (B) PL spectra of monolayer MoS_2_ before and after the etching process. (C) Transfer characteristics and (D) output characteristics of monolayer MoS_2_ edge-contact FET under different etching conditions with *L*_ch_ = 1 μm. The back-gate voltages range from *V*_bg_ = −0.5 V to *V*_bg_ = 5.5 V in 0.5-V step.

In addition to the direct observation of the microscopic morphology, we further characterized the material properties of monolayer MoS_2_ before and after the etching process for the edge-contact structure by spectroscopic analysis, which was used to confirm that the monolayer MoS_2_ in the contact regions had been completely removed prior to contact metallization. Raman and photoluminescence (PL) spectra of the monolayer MoS_2_ in the contact regions were characterized before and after the etching process, as depicted in Fig. 2A and B. As shown by the black line in Fig. 2A, the Raman spectra exhibit the E^1^_2g_ and A_1g_ peaks with a position difference of 19.5 cm^−1^, confirming that the as-grown MoS_2_ is monolayer, consistent with previous reports [39,40]. The Raman spectrum of the etched monolayer MoS_2_ in the contact regions completely disappeared from the E^1^_2g_ and A_1g_ mode after the etching process. Similarly, the PL spectrum of the etched monolayer MoS_2_ in the contact regions was also completely absent after the etching process, indicating that monolayer MoS_2_ in the contact region had been fully removed.

In order to visually understand the electrical transport properties of edge contacts constructed under different etching conditions, the 2 batches of devices were compared side by side, except that different etching processes (Ar plasma or O_2_/Ar plasma) were used. Figure 2C shows the transfer characteristics (*I*_d_–*V*_g_ curves) of the edge-contact monolayer MoS_2_ transistors with *L*_ch_ = 1 μm at *V*_ds_ = 0.1 V by using the O_2_/Ar plasma and Ar plasma process, respectively. The edge-contact monolayer MoS_2_ FET by using Ar plasma shows a 10 times higher on-state current than by using O_2_/Ar plasma at the same channel length, which may be attributed to the oxidation of the edges of the etched MoS_2_ by using O_2_/Ar plasma. The output characteristics (*I*_d_–*V*_d_ curves) of the transistors are shown in Fig. 2D. The edge-contact monolayer MoS_2_ FET by using Ar plasma exhibits ohmic contact behavior with *I*_ds_ of 97 μA/μm at *V*_ds_ = 1 V, while the device using O_2_/Ar plasma exhibits Schottky behavior with *I*_ds_ of 8 μA/μm at *V*_ds_ = 1 V. The electrical transport properties of devices fabricated under O_2_/Ar etching conditions deteriorated substantially, confirming the significant impact of the channel edge dangling bonds on the edge-contact quality.

**Fig. 3.**
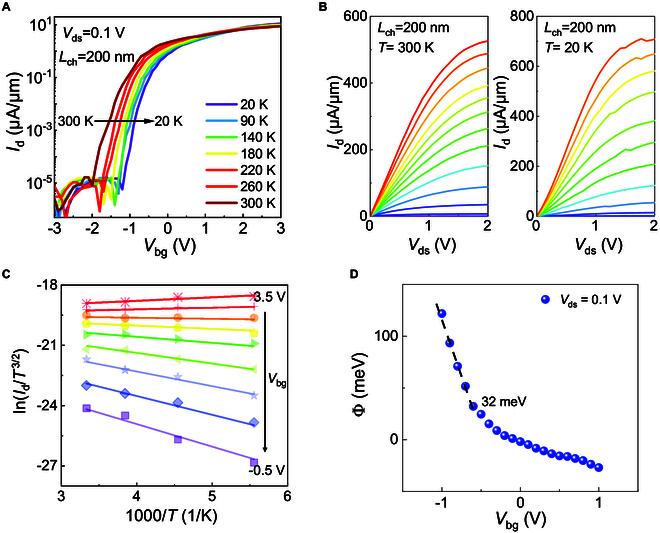
Low-temperature measurements for monolayer MoS_2_ edge-contact FET. (A) Transfer characteristics of monolayer MoS_2_ edge-contact FET with temperature from 300 to 20 K with *L*_ch_ = 200 nm. (B) Output characteristics of monolayer MoS_2_ edge-contact FET at 300 and 20 K with *L*_ch_ = 200 nm. The back-gate voltages range from *V*_bg_ = −0.5 V to 6 V in 0.5-V step. (C) Arrhenius plot of drain current at various back-gate voltages for calculations of the Schottky barrier heights. (D) Schottky barrier extraction from edge-contact FET.

To further examine the edge-contact quality for the Ar plasma process, we performed low-temperature measurements of edge-contact FETs with a 200-nm short channel. We present the transfer characteristics of devices with *L*_ch_ = 200 nm from 300 K to 20 K, as shown in Fig. 3A. It can be observed that the *I*_d_–*V*_g_ curves at different temperatures cross around *V*_bg_ = 1.7 V, which is common in 2D devices and illustrates the transition of the channel from insulating to metallic properties with gate control. Figure 3B compares the output characteristic curves of the edge-contact device with *L*_ch_ = 200 nm at room temperature of 300 K and cryogenic temperature of 20 K (see Fig. S2). The monolayer MoS_2_ edge-contact FET exhibited a linear output curve with a high output currents of 380 and 525 μA/μm at supplied drain–source voltage of 1 V and 2 V, respectively, demonstrating ohmic contact at room temperature. It is clearly shown that the saturated output current density increases from 524 μA/μm at 300 K to 710 μA/μm at 20 K under the same voltage bias of *V*_ds_ = 2 V and *V*_bg_ = 6 V, accompanied by current saturation. As depicted in Fig. 3B, the output characteristic curves remain nearly linear even at 20 K, demonstrating quasi-ohmic contact. The low-temperature quasi-ohmic contact phenomenon observed in our FETs is similar to that seen in other top contacts, such as graphene contacts [45] and h-BN/MoS_2_ stacks contacts [46], and is crucial to achieving high electrical performance. The Schottky barrier height (SBH; Φ_B_) was extracted using the thermionic emission equation described below.

$$I_{d}=A_{2D}^{*}T^{2/3}\exp\left(-\frac{q\Phi_{B}}{k_{B}T}\right)\left[1-\exp\left(\frac{qV_{\mathrm{ds}}}{k_{B}T}\right)\right]$$

**Fig. 4.**
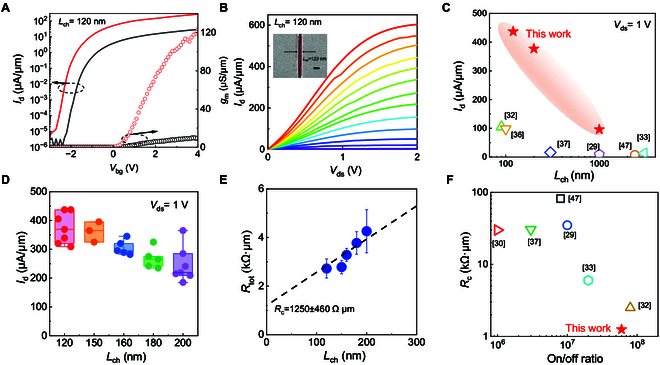
The electrical characteristics of monolayer MoS_2_ edge-contact FETs. (A) Transfer characteristics and transconductance of monolayer MoS_2_ edge-contact FET with *L*_ch_ = 120 nm at *V*_ds_ = 1 V (red) and *V*_ds_ = 0.1 V (black). (B) Output characteristics of monolayer MoS_2_ edge-contact FET with *L*_ch_ = 120 nm. The back-gate voltages range from *V*_bg_ = −0.5 V to 6 V in 0.5-V step. The inset figure is the corresponding SEM image of device for channel length measurement. Inset SEM image scale bar, 200 nm. (C) Output characteristics of monolayer MoS_2_ edge-contact FET with *L*_ch_ = 120 nm. Output current benchmark of monolayer MoS_2_ edge-contact FETs with channel length dependence at *V*_ds_ = 1 V. (D) Statistics of output current of monolayer MoS_2_ edge-contact FETs with different *L*_ch_ at *V*_ds_ = 1 V. (E) Contact resistance extracted for monolayer MoS_2_ edge-contact FETs with different *L*_ch_. (F) Contact resistance and on/off ratio benchmark of monolayer MoS_2_ edge-contact FETs.

In this equation, *I*_d_ is the drain–source current, *A*^∗^_2D_ is the 2D equivalent Richardson constant, *T* is the temperature, *k*_B_ is the Boltzmann constant, *q* is the electronic charge, and *V*_ds_ is the drain–source voltage bias. As shown in Fig. 3C, as the back-gate voltage decreases, the slope of ln(*I*_d_/*T*^3/2^) versus 1,000/*T* on the Arrhenius plot changes from negative to positive. The extracted SBH is as low as 32 meV at a drain–source voltage bias of 0.1 V, as shown in Fig. 4D. The low SBH in the edge-contacted FETs promotes excellent electrical performance.

To evaluate the potential of edge**-**contact monolayer MoS_2_ transistors at scaled channel lengths, a 120-nm short-channel high-performance edge-contact FET was demonstrated. The corresponding scanning electron microscopy (SEM) image is shown in the inset of Fig. 4B. Figure 4A shows the transfer characteristics of the edge-contact monolayer MoS_2_ FET at *V*_ds_ = 0.1 and 1 V. The device exhibits high on-state current, low sub-threshold swing (SS) of 150 mV/dec, and an on/off ratio of 6 × 10^7^. Owing to the low SBH of the edge contacts and high-quality HfO_2_ dielectric layer for MoS_2_ transistors, the peak transconductance *g*_m_ of the device reaches 123 μS/μm at *V*_ds_ = 1 V. As shown in the output characteristic curves of Fig. 4B, high output current of 605 and 436 μA/μm is obtained at supplied drain–source voltage of 2 and 1 V, respectively. To the best of our knowledge, the output current of 436 μA/μm at *V*_ds_ = 1 V in this work represents the highest output current density for monolayer MoS_2_ edge-contact FETs to date, more than 4 times higher than previous studies as shown in benchmark figure of Fig. 4C. Table S1 lists the specific comparisons of different electrical parameters. Furthermore, through statistical analysis of the measurement results of several edge-contact devices with different channel lengths (*L*_ch_ = 120, 150, 160, 180, and 200 nm), the repeatability of high output current in short-channel edge-contact FETs at *V*_ds_ = 1 V can be confirmed, as shown in Fig. 4D. Obviously, the output current of all edge-contact devices remains at a high level, verifying the high-quality edge contacts in this work.

Essentially, the high output current and on-state current of edge-contact FET can be attributed to the low *R*_c_ generated by the well-constructed bonded edge interface. By extensively analyzing the output current with different channel lengths, the *R*_c_ for the edge-contact FETs is extracted as shown in Fig. 4E. Contact resistance extracted from devices with channel lengths below 200 nm is more accurate, as the contact resistance either dominates the total resistance or is at least comparable to the channel resistance. The *R*_c_ value of edge-contact monolayer MoS_2_ FETs is 1.25 ± 0.46 kΩ·μm, better than the values of edge-contact MoS_2_ FETs reported in the literatures [29,30,32,33,37,47], as depicted in Fig. 4F. Furthermore, a scaled contact length (*L*_c_) device with *L*_c_ = 120 nm and *L*_ch_ = 220 nm was also successfully demonstrated experimentally to show the potential of device scaling (Fig. S3).

## Discussion

We have reported the high-performance edge-contact monolayer MoS_2_ FET by using CVD grown monolayer MoS_2_, with channel length reduced to 120 nm. The plasma etching by pure Ar improves the edge dangling bonds and thus improves the edge-contact quality. Edge-contact monolayer MoS_2_ FETs exhibit good ohmic contact at both room temperature and cryogenic temperatures, and have a record-low contact resistance of 1.25 kΩ·μm among all edge-contact monolayer MoS_2_ FETs. Record-high output current of 436 μA/μm and transconductance of 123 μS/μm with a steep SS of 150 mV/dec are achieved on an edge-contact monolayer MoS_2_ FET with *L*_ch_ = 120 nm on 9-nm HfO_2_ dielectric. For edge contacts, the core advantage is that it can be immune to the deterioration of performance caused by the reduction of top contact length and can build stacked nanosheet structures, which is crucial for achieving multi-layer TMD channel stacked device structures to break through the physical gate length limitations of traditional semiconductor devices while improving device performance. Due to the limitation of experimental equipment and materials, the width of source–drain metal electrodes demonstrated in this work needs to be further reduced to 10 nm in the future, edge-contact devices for multi-bridge channels need to be explored and validated, and device reliability under this structure needs to be further explored. In addition, novel metallic materials and deposition techniques can also be employed in subsequent studies to achieve further reduction of contact resistance and achieve the robustness of the device’s mechanical structure.

## Materials and Methods

### CVD growth of monolayer MoS_2_ and material characterization

Monolayer MoS_2_ was grown on molten glass. The growth was carried out using a furnace with 2 temperature zones, with temperatures of 250 and 860 °C in each zone. The sulfur powder was loaded onto the quartz boat and placed in zone I, while the MoO_3_ precursor and the molten glass were loaded into a porcelain boat and placed in zone II. Ar gas was always supplied during the growth process. At 860 °C, the growth time of MoS_2_ was about 35 min. MoS_2_ was transferred to the target substrate through wet method. The basic morphology of MoS_2_ was observed using an optical microscope. The thickness of MoS_2_ was basically determined by Raman and PL.

### FET fabrication

The grown MoS_2_ was transferred by wet method onto the high-quality HfO_2_ dielectric deposited by atomic layer deposition. Generally, the thickness of HfO_2_ used is about 9 nm. To obtain a regular strip-shaped MoS_2_, the electron beam lithography (Raith eLine) and Ar/CF_4_ plasma etching (by reactive ion etching) were used. Subsequently, monolayer MoS_2_ was annealed at 250 °C for 1 h in Ar atmosphere. After electron beam lithography and etching, monolayer MoS_2_ in the contact area was removed by reactive ion etching. The contact metal (Ni/Au) was then deposited under high vacuum conditions.

### Electrical measurement

The direct-current electrical measurements were conducted using a Lakeshore probe station and an Agilent B1500A semiconductor parameter analyzer. All measurements are conducted in a vacuum of 10^−4^ torr to reduce the influence of moisture and oxygen in the measurement environment.

## Data Availability

All data required to support the conclusions are presented in the main text and the Supplementary Materials.
