# Supplementary material for: High-Performance Edge-Contact Monolayer Molybdenum Disulfide Transistors
Source: Research (Wash D C). 2025 Jan 17;8:0593. doi: 10.34133/research.0593 (PMC11739435; doi:10.34133/research.0593)
Supplement: Supplementary 1 — Figs. S1 to S3 Table S1 [file research.0593.f1.docx]

**Supplementary Material**

**High-Performance Edge-Contact Monolayer Molybdenum Disulfide Transistors**

Jiankun Xiao^1^, Xiong Xiong^1^*, Xinhang Shi^2^, Shiyuan Liu^1^, Shenwu Zhu^2^, Yue Zhang^3^, Ru Huang^1^ and Yanqing Wu^1,2^*

^1^School of Integrated Circuits and Beijing Advanced Innovation Center for Integrated Circuits, Peking University, Beijing 100871, China.

^2^Wuhan National High Magnetic Field Center and School of Integrated Circuits, Huazhong University of Science and Technology, Wuhan 430074, China.

^3^Academy for Advanced Interdisciplinary Science and Technology, Beijing Advanced Innovation Center for Materials Genome Engineering, University of Science and Technology Beijing, Beijing 100083, China

*Address correspondence to: [xiongxiong@pku.edu.cn](mailto:xiongxiong@pku.edu.cn), [yqwu@pku.edu.cn](mailto:yqwu@pku.edu.cn)

**Fig. S1. Schematic diagram of key process flow for edge contact monolayer MoS_2_ FETs fabrication.**

Fig. S1. Device fabrication process flow for edge-contact monolayer MoS_2_ FETs in this work. The process details are also provided in the Methods section.

**Fig. S2.** **Temperature dependence of electrical performance of 200-nm monolayer MoS_2_ edge-contact FETs.**


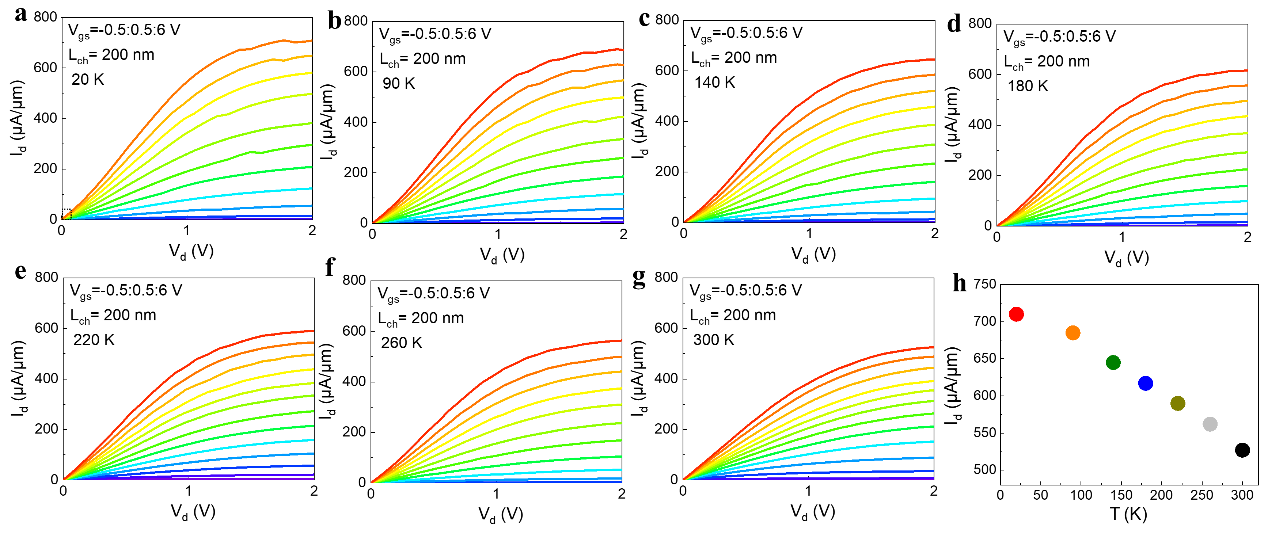


Fig. S2. (a-f) Output characteristics of monolayer MoS_2_ edge contact FET with temperature from 20 to 300 K with L_ch_=200 nm. (f) Summary of output current at different temperatures at V_d_=2 V.

**Fig.S3. Monolayer MoS_2_ edge contact FETs with contact length of 120 nm.**


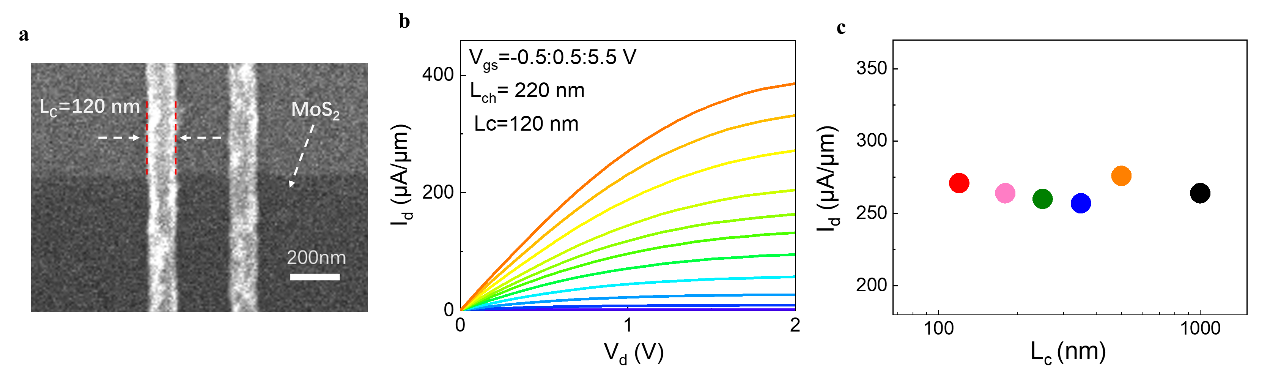


Fig. S3. (a) SEM image of monolayer MoS_2_ edge contact FET with small L_c_ =120 nm. Scale bar, 200 nm. (b) Output characteristics of monolayer MoS_2_ edge contact FET with L_c_=120 nm.

**Table S1. Benchmark table for edge contact MoS_2_ FETs.**

| **Ref** | **thickness** | **L_ch_**  **(nm)** | **g_m_**  **(μS/μm)** | **R_C_ (kΩ·μm)** | **I_on_@V_d_=1 V (μA/μm)** | **On/off**  **ratio** |
| --- | --- | --- | --- | --- | --- | --- |
| **This**  **Work** | **1L** | **120** | **123** | **1.25** | **436** | **6×10^7^** |
| 29 | 1L | 1000 | NA | 35 | 9 | 3×10^7^ |
| 30 | 1L | NA | NA | 30 | NA | 10^6^ |
| 32 | 1L | 90 | NA | 2.5 | 110 | 8×10^7^ |
| 33 | ML | 3000 | NA | 6.1 | 12 | 2×10^7^ |
| 36 | 1L | 100 | NA | NA | 100 | NA |
| 37 | 1L | 300 | NA | 30.5 | 15 | 3×10^6^ |
| 47 | 1L | 2400 | NA | 82(ML) | 2 | 3×10^6^ |
